# Retraction of “Artificial Intelligence in Natural Product Drug Discovery: Current Applications and Future Perspectives”

**DOI:** 10.1021/acs.jmedchem.5c03333

**Published:** 2026-01-05

**Authors:** Amit Gangwal, Antonio Lavecchia

The authors retract this Perspective (DOI: 10.1021/acs.jmedchem.4c01257) due to insufficient attribution and unacceptable overlap with previously published content. An Addition/Correction was posted on June 26, 2025 (DOI: 10.1021/acs.jmedchem.5c01675) to properly acknowledge a prior comprehensive review on the same topic (DOI: 10.1038/s41573-023-00774-7); however, further concerns have been identified, and the authors agreed with the Editor-in-Chief that the text overlap between the two articles is excessive. As a result, the article is being retracted.

The original article was published on February 6, 2025, corrected on June 26, 2025, and retracted on January 5, 2026.

